# Relationship Between Short‐Term Outcomes and PD‐L1 Expression Based on Combined Positive Score and Tumor Proportion Score in Recurrent or Metastatic Head and Neck Cancers Treated With Anti‐PD‐1 Antibody Monotherapy

**DOI:** 10.1002/cnr2.70125

**Published:** 2025-01-22

**Authors:** Akihiro Ohara, Taisuke Mori, Mai Itoyama, Kazuki Yokoyama, Shun Yamamoto, Ken Kato, Yoshitaka Honma

**Affiliations:** ^1^ Department of Head and Neck, Esophageal Medical Oncology National Cancer Center Hospital Tokyo Japan; ^2^ Department of Diagnostic Pathology National Cancer Center Hospital Tokyo Japan

**Keywords:** biomarkers, chemotherapy, head and neck cancer, prognostic factor

## Abstract

**Background:**

PD‐L1 expression in tumors and immune cells is a biomarker for the efficacy of anti‐PD‐1 antibody (APA) therapy across diverse cancers. Based on the results from the KEYNOTE‐048 trial, pembrolizumab monotherapy is indicated for platinum‐sensitive recurrent/metastatic head and neck squamous cell carcinoma (R/M‐HNSCC) with a positive combined positive score (CPS). Conversely, nivolumab is utilized for platinum‐pretreated R/M‐HNSCC regardless of the positive tumor proportion score (TPS) following the results of the CheckMate‐141; however, its subgroup analysis indicated that TPS‐positive population tended to have a relatively high overall response rate and progression‐free survival (PFS). Although, the superior PD‐L1 evaluation method for predicting APA therapy efficacy in R/M‐HNSCC and the appropriate cut‐off value remain undetermined. This study aims to elucidate the relationship between short‐term outcomes and PD‐L1 expression based on CPS and TPS in R/M‐HNSCC patients undergoing APA monotherapy.

**Methods:**

R/M‐HNSCC patients receiving APA monotherapy from 2018 to 2021 with available samples were enrolled. An experienced pathologist evaluated CPS and TPS utilizing the PD‐L1 IHC 22C3 pharmDx assay. Short‐term outcomes were assessed by clinical benefit rate (CBR), objective response rate (ORR), and PFS.

**Results:**

Fifty‐three R/M‐HNSCC patients received APA monotherapy. Forty‐seven had CPS ≥ 1, and 44 had TPS ≥ 1%. By receiver‐operating characteristic curve analysis, the CPS cut‐off value for predicting better CBR was determined to be 50. The ORR/CBR tended to be higher when CPS was positive. Although differences in PFS were not observed for a cut‐off value of 1 or 20, they were observed for 50 (3.2 vs. 8.4 months; hazard ratio 0.44, *p* = 0.02). ORR and CBR were respectively 12.5% and 12.5% in the TPS < 1% group and 33.3% and 48.9% in the ≥ 1% group. The TPS < 1% group showed significantly poorer PFS (1.9 vs. 4.5 months, hazard ratio 0.40, *p* = 0.01).

**Conclusion:**

The short‐term efficacy of APA monotherapy in R/M‐HNSCC patients tended to be better when CPS was positive. TPS helps predict the population that does not benefit from APA monotherapy.

## 1 Introduction

Expression of programmed death ligand 1 (PD‐L1) in tumor tissue predicts the clinical benefit of agents that block programmed cell death protein 1 (PD‐1) in cancer patients. The combined positive score (CPS) and tumor proportion score (TPS) are widely used to measure PD‐L1 expression. Several reports on various types of cancer suggest that CPS is more sensitive than TPS in predicting responsiveness to anti‐PD‐1 or anti‐PD‐L1 antibodies [1, 2].

Pembrolizumab and nivolumab are monoclonal antibodies of the immunoglobulin G4 class targeting PD‐1, which inhibit the interaction between PD‐1 and its ligands, PD‐L1 and programmed death ligand 2. This inhibition leads to the prevention of activated T‐cell suppression, resulting in anti‐tumor effects. In the KEYNOTE‐048 trial, pembrolizumab was clinically effective in patients with platinum‐sensitive recurrent or metastatic head and neck squamous cell carcinoma (SCC) [3]. The CheckMate 141 trial also demonstrated a survival benefit of nivolumab in patients with platinum‐refractory recurrent or metastatic head and neck SCC (R/M‐HNSCC) [4]. In the KEYNOTE‐048 trial, overall survival was significantly better on pembrolizumab monotherapy than on cetuximab with platinum and 5‐fluorouracil in populations with CPS ≥ 1 or CPS ≥ 20 [3]. In contrast, nivolumab monotherapy for platinum‐pretreated R/M‐HNSCC tended to achieve better clinical outcomes in the population with TPS ≥ 1% than in the population with TPS < 1% in the CheckMate 141 [4]. These two trials evaluated the expression of PD‐L1 using different methods, and the CPS cut‐off value has been set at only 1 and 20 based on the results of the KEYNOTE‐048 trial. Therefore, it is still unclear which PD‐L1 expression measurement method is more sensitive and whether the currently used CPS cut‐off value best predicts the clinical outcome in R/M‐HNSCC patients treated with APA monotherapy.

The purpose of this study is to clarify the clinical outcomes according to CPS and TPS status and the appropriate CPS cut‐off value for the prediction of short‐term outcomes in patients with R/M‐HNSCC treated by APA monotherapy.

## 2 Patients and Methods

### 2.1 Patient Characteristics

Patients with HNC in whom CPS was evaluated between January 2018 and December 2021 at the National Cancer Center Hospital were screened. Data regarding age, sex, Eastern Cooperative Oncology Group performance status, clinical characteristics, and clinical outcomes for patients with R/M‐HNSCC who received APA monotherapy was collected from the medical records. The study received approval from the institutional ethics committee at the National Cancer Center Hospital (2018‐168). Written informed consent was obtained from all patients.

### 2.2 Evaluation of PD‐L1 Expression in Tumor Samples

The experienced pathologist, without access to clinical data, and the same physician assessed PD‐L1 expression in formalin‐fixed, paraffin‐embedded specimens. The samples collected closest to the time of administration of APA monotherapy were used regardless of the timing of sample collection. Immunohistochemical staining was conducted utilizing the PD‐L1 IHC 22C3 pharmDx assay (Dako, Glostrup, Denmark).

Methods for measuring CPS in clinical practice were established following the KEYNOTE‐048 trial [3]. The US Food and Drug Administration and the European Medicines Agency have endorsed pembrolizumab, encompassing the CPS measurement technique. CPS was obtained by dividing the number of PD‐L1 positive cells by the total number of tumor cells and multiplying the result by 100. PD‐L1 positive cells were counted within tumor cells, lymphocytes, and macrophages. However, different TPS measurement methods were used in the CheckMate 141 and the KEYNOTE‐040 trials, and which is best is controversial [4, 5]. Therefore, actual values were calculated for CPS, but only positive or negative values were assessed for TPS in this study.

### 2.3 Statistical Analysis

The Chi‐squared or Fisher's exact test assessed differences in categorical variables. The tumor response was evaluated following the Response Evaluation Criteria for Solid Tumors version 1.1. Clinical benefit rate (CBR) was defined as the proportion of patients showing stable disease for at least 6 months, a partial response, and a complete response. Progression‐free survival (PFS) was calculated from the commencement of APA monotherapy to disease progression, death, or the last follow‐up visit, and it was estimated utilizing the Kaplan–Meier method. Survival was assessed through comparison using the log‐rank test. The appropriate CPS cut‐off values for predicting better clinical outcomes of APA monotherapy in patients with R/M‐HNSCC were determined by receiver‐operating characteristic curve analysis, and the optimal cut‐off value was selected using the Youden index. The data were analyzed using JMP Pro version 14.2.0 (SAS Institute Inc., Cary, NC, USA). A *p*‐value < 0.05 was considered statistically significant.

## 3 Results

### 3.1 Patient Characteristics

**FIGURE 1:**
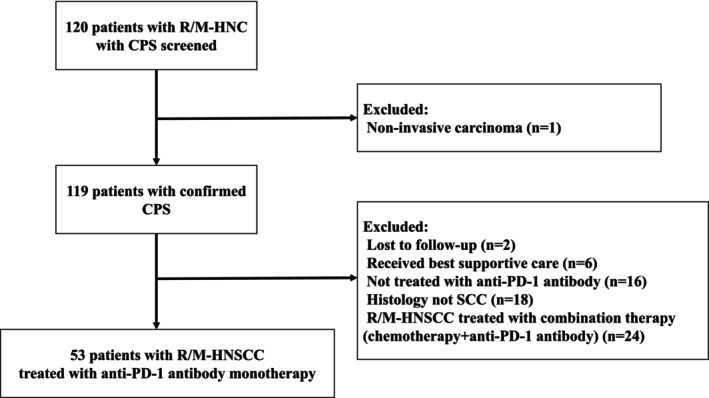
CONSORT diagram. CPS, combined positive score; PD‐1, programmed cell death protein 1; RM‐HNS, recurrent/metastatic head and neck cancer; R/M‐HNSCC, recurrent/metastatic head and neck squamous cell carcinoma; SCC, squamous cell carcinoma.

We identified 119 patients with HNC for whom tumor samples were available (Figure 1). SCC was the most common histological type, and patients with SCC had a higher rate of PD‐L1 expression positivity than those without SCC (CPS, 85.1% vs. 50.0%, *p* < 0.001; TPS, 81.2% vs. 38.9%, *p* < 0.001; Tables 1 and 2). Patients with salivary gland cancer (SGC) were less likely than those without SGC to show positive PD‐L1 expression (CPS, 55.6% vs. 81.8%, *p* = 0.08; TPS, 44.4% vs. 77.3%, *p* = 0.04). The PD‐L1 positivity expression rate was low in acinic cell carcinoma and adenoid cystic carcinoma, which are derived from major or minor salivary glands.

**TABLE 1:** Pathological characteristics associated with PD‐L1 expression in patients with head and neck cancer.

	Total (*N* = 119)	TPS		CPS			
		< 1	≥ 1	< 1	1 to ≤ 20	20 to < 50	≥ 50
Primary tumor location, Histological type							
Hypopharynx (*n* = 36, 30.3%)							
SCC	36	11	25	9	15	5	7
Oropharynx (*n* = 20, 16.8%)							
SCC	20	4	16	2	11	4	3
Nasopharynx (*n* = 4, 3.4%)							
SCC	4	1	3	1		1	2
Larynx (*n* = 2, 1.7%)							
SCC	2		2			2	
Oral cavity (*n* = 26, 21.8%)							
SCC	24	2	22	2	6	7	9
Malignant melanoma	1		1			1	
Mucoepidermoid carcinoma	1	1		1			
Salivary gland (*n* = 9, 7.6%)							
SCC	3		3			2	1
Adenoid cystic carcinoma	2	2		2			
Adenocarcinoma	2	1	1		1	1	
Acinic cell carcinoma	1	1		1			
Myoepithelial carcinoma	1	1		1			
Nasal cavity, Paranasal sinuses (*n* = 12, 10.1%)							
SCC	7	1	6	1	2		4
Adenoid cystic carcinoma	2	1	1	1	1		
Adenocarcinoma	2	1	1	1	1		
Sinonasal undifferentiated carcinoma	1	1			1		
External auditory canal (*n* = 4, 3.4%)							
SCC	3		3		1	1	1
Adenoid cystic carcinoma	1	1		1			
Unknown (*n* = 4, 3.4%)							
SCC	2		2		1		1
Carcinosarcoma	1	1		1			
Mucoepidermoid carcinoma	1		1			1	
Eyelid (*n* = 1, 0.8%)							
Sebaceous carcinoma	1		1		1		
Scalp (*n* = 1, 0.8%)							
Sebaceous carcinoma	1		1		1		

Abbreviations: CPS, combined positive score; HNC, head and neck cancer; PD‐L1, programmed death ligand 1; SCC, squamous cell carcinoma.

**TABLE 2:** Relationship between histological type of head and neck cancer and PD‐L1 expression.

	Total (*N* = 119)	TPS		CPS			
		< 1	≥ 1	< 1	≥ 1 to < 20	≥ 20 to < 50	≥ 50
SCC	101	19	82	15	36	22	28
Adenocarcinoma, NOS	4	2	2	1	2	1	
ACC	1	1		1			
AdCC	5	4	1	4	1		
Carcinosarcoma	1	1		1			
Malignant melanoma	1		1			1	
Mucoepidermoid carcinoma	2	1	1	1		1	
Myoepithelial carcinoma	1	1		1			
Sebaceous carcinoma	2		2		2		
Sinonasal undifferentiated carcinoma	1	1			1		

Abbreviations: ACC, acinic cell carcinoma; AdCC, adenoid cystic carcinoma; CPS, combined positive score; HNC, head and neck cancer; NOS, not otherwise specified; PD‐L1, programmed death ligand 1; SCC, squamous cell carcinoma.

All TPS‐positive patients were CPS‐positive, but 6 CPS‐positive patients were TPS‐negative. Five TPS‐negative patients had low CPS (1 or 5) and one had high CPS (20).

### 3.2 Clinical Outcomes of R/M‐HNSCC Treated With APA Monotherapy

Twenty‐six of the 53 patients with R/M‐HNSCC treated using APA monotherapy received pembrolizumab and 27 received nivolumab (Figure 1). Their characteristics are shown in Table 2. In the overall population, the ORR was 30.2% (95% confidence interval [CI] 19.5–43.5) and the CBR was 43.4% (95% CI 31.9%–56.7%). There were no significant differences in ORR and CBR between the pembrolizumab and nivolumab groups (23.1% [95% CI 11.0–42.1] and 46.2% [95% CI 28.8%–64.5%], respectively, in the pembrolizumab group and 37.0% [95% CI 21.5–55.8] and 40.7% [95% CI 24.5%–59.3%], respectively, in the nivolumab group [ORR, *p* = 0.37; CBR, *p* = 0.78]; Table 3).

**TABLE 3:** Characteristics of patients who received anti‐PD‐1 antibody monotherapy.

	Overall population *N* = 53	Pembrolizumab *n* = 26	Nivolumab *n* = 27
Age, years	69 (30–83)	71 (30–83)	69 (36–78)
Sex, *n* (%)			
Female	8 (15.1)	4 (15.4)	4 (14.8)
Male	45 (84.9)	22 (84.6)	23 (85.2)
ECOG‐PS, *n* (%)			
0	36 (67.9)	18 (69.2)	18 (66.7)
1	16 (30.1)	8 (30.8)	8 (29.6)
2	1 (1.9)	0	1 (3.7)
Treatment line, *n* (%)			
1	13 (24.5)	13 (50.0)	0
2	30 (56.6)	9 (34.6)	21 (77.8)
≥ 3	10 (18.9)	4 (15.4)	6 (22.2)
CPS, *n* (%)			
TPS < 1	9 (17.0)	2 (7.7)	7 (25.9)
TPS ≥ 1	44 (83.0)	24 (92.3)	20 (74.1)
CPS < 1	6 (11.3)	2 (7.7)	4 (14.8)
CPS ≥ 1 to < 20	20 (37.7)	9 (34.6)	11 (40.7)
CPS 20 to < 50	12 (22.6)	6 (23.1)	6 (22.2)
CPS ≥ 50	15 (28.3)	9 (34.6)	6 (22.2)
Primary tumor location, *n* (%)			
Hypopharynx	22 (41.5)	9 (34.6)	13 (48.1)
Oropharynx	12 (22.6)	7 (26.9)	5 (18.5)
Nasopharynx	4 (7.5)	3 (11.5)	1 (3.7)
Larynx	1 (1.9)	0	1 (3.7)
Oral cavity	8 (15.1)	4 (15.4)	4 (14.8)
Nasal cavity, Paranasal sinuses	2 (3.8)	1 (3.8)	1 (3.7)
External auditory canal	2 (3.8)	1 (3.8)	1 (3.7)
Unknown primary	2 (3.8)	1 (3.8)	1 (3.7)
Prior surgery, *n* (%)	35 (66.0)	16 (61.5)	19 (70.4)
Prior radiotherapy, *n* (%)	41 (77.4)	21 (80.8)	20 (74.1)

Abbreviations: CPS, combined positive score; ECOG‐PS, Eastern Cooperative Oncology Group performance status; PD‐1, programmed cell death protein 1; TPS, tumor proportion score.

**FIGURE 2:**
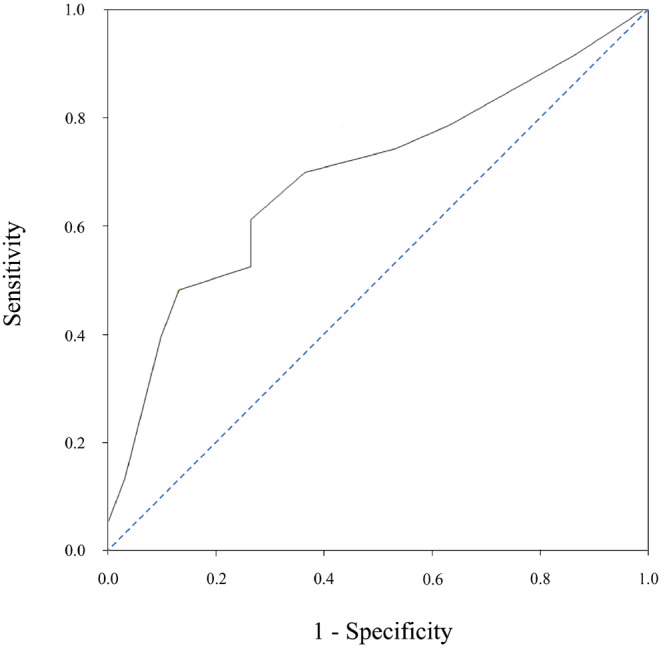
Receiver‐operating characteristic curve analysis of CPS for predicting better clinical outcomes of APA monotherapy (cut‐off = 50.0, AUC = 0.69).

**FIGURE 3:**
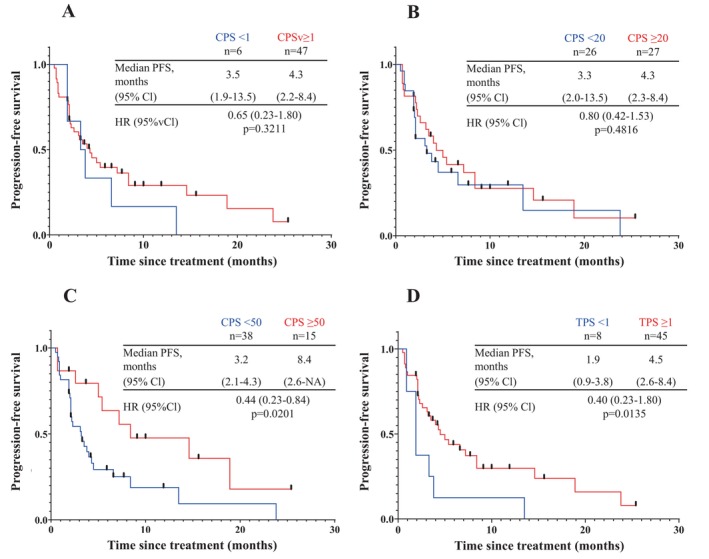
Progression‐free survival when the CPS cut‐off value is (A) 1, (B) 20, or (C) 50 and the TPS cut‐off value is (D) 1 in patients treated with anti‐programmed death ligand‐1 monotherapy. CI, confidence interval; CPS, combined positive score; HR, hazard ratio; NA, not available; PFS, progression‐free survival; PS, tumor proportion score; TPS, tumor proportion score.

The ORR and CBR for APA monotherapy were, respectively, 16.7% (95% CI 3.0–56.4) and 33.3% (95% CI 9.7–70.0) for CPS < 1, 31.9% (95% CI 20.4–46.2), and 44.7% (95% CI 31.4–58.8) for CPS ≥ 1, and 40.7% (95% CI 24.5–59.3) and 59.3% (95% CI 40.7–75.5) for CPS ≥ 20. When using receiver‐operating characteristic curve analysis (Figure 2), the CPS of 50 was established as the threshold for predicting an improved CBR (area under the curve, 0.69; sensitivity, 0.48; specificity, 0.87). The ORR and CBR were, respectively, 46.7% (95% CI 24.8–69.9) and 73.3% (95% CI 48.0–89.1) for CPS ≥ 50. Although no differences in PFS were observed when the CPS cut‐off value was set to 1 or 20 (Figure 3), a significant difference was observed when the CPS cut‐off value was set to 50 (CPS < 50 vs. CPS ≥ 50: 3.2 [95% CI 2.1–4.3] months vs. 8.4 [95% CI 2.6 to not available] months, hazard ratio [HR] 0.44, *p* = 0.02). The upper bound of the 95% CI was not available for CPS ≥ 50 due to the small sample size, which limited the ability to estimate the entire range.

The ORR and CBR were both 12.5% (95% CI 2.2–47.1) for TPS < 1% and were 33.3% (95% CI 21.4–47.9) and 48.9% (95% CI 35.0–63.0), respectively, for TPS ≥ 1%. Median PFS was significantly worse in the TPS < 1% group (TPS < 1% vs. TPS ≥ 1%: 1.9 [95% CI 0.9–3.8] months vs. 4.5 [95% CI 2.6–8.4] months, HR 0.40, *p* = 0.01).

## 4 Discussion

This study found histological variations in PD‐L1 expression across HNC. In the KEYNOTE‐048 trial [3], the respective percentages of the R/M‐HNSCC population with CPS ≥ 1 and CPS ≥ 20 were 85% and 43%, and these findings are consistent with those in our study. As far as we have investigated, there have been no reports on differences in PD‐L1 expression between SCC and other histological types in HNC. Several studies have compared SCC with adenocarcinoma in the lung and cervical cancer [6, 7, 8, 9], with SCC generally exhibiting higher levels of PD‐L1 expression. The histological types of SGC are diverse, and several studies have assessed histology‐specific PD‐L1 expression [10, 11, 12, 13]. Salivary duct carcinoma appears to have a favorable immunogenic profile characterized by a moderate tumor mutation burden and medium or high inflammation in the microenvironment [14], and several studies have shown that salivary duct carcinoma has high PD‐L1 expression [15, 16, 17, 18, 19, 20, 21]. Several studies have associated high PD‐L1 expression with poor prognosis, particularly when expressed on tumor cells rather than immune cells [21]. High PD‐L1 expression is also detected in high‐grade mucoepidermoid carcinoma, SCC, and adenocarcinoma, not otherwise specified, among other high‐grade SGCs [10, 11, 12, 18, 20, 22]. The tumor microenvironment in adenoid cystic carcinoma may be less immunogenic. It is characterized by a low tumor mutation burden, few tumor‐infiltrating lymphocytes, low dendritic cell density, and a high frequency of human leucocyte antigen class I deficiency [15, 23, 24]. Therefore, PD‐L1 expression may be lower in low‐grade SGCs such as adenoid cystic carcinoma than in high‐grade SGCs. PD‐L1 expression was elevated in SCC and adenocarcinoma compared to adenoid cystic carcinoma and acinic cell carcinoma, aligning with previous research findings.

Although our study found no significant difference in CBR or PFS at a CPS cut‐off value of 1 or 20, a clinically meaningful difference was demonstrated at a CPS cut‐off value of 50. In the KEYNOTE‐048 trial, PFS was no better on pembrolizumab monotherapy than on cetuximab‐based chemotherapy, even in the CPS ≥ 20 group (HR 0.99, 95% CI 0.75–1.29, *p* = 0.4562). However, in the post hoc analyses of the KEYNOTE‐040 trial, which compared pembrolizumab with the investigator's choice of treatment (methotrexate, docetaxel, or cetuximab) in patients with platinum‐pretreated R/M‐HNSCC, the difference in PFS was not significant in the CPS < 50 group but was significant in the CPS ≥ 50 group [2]. These findings indicate that the benefit from APA monotherapy is greater for higher CPS.

In the CheckMate 141, which demonstrated the superiority of nivolumab over the investigator's choice of treatment (methotrexate, docetaxel, or cetuximab) in patients with platinum‐refractory R/M‐HNSCC, subgroup analyses showed that the clinical outcome tended to be better in patients on nivolumab in the TPS ≥ 1% group than in the TPS < 1% group. The 2‐year follow‐up data from the CheckMate 141 showed an overall survival benefit in the TPS ≥ 1% group, with a consistent 45% decrease in the risk of death when compared to the investigator's choice of treatment (median overall survival: 8.2 [95% CI 6.7–9.5] months vs. 4.7 [95% CI3.8–6.2] months, HR 0.55 [95% CI 0.39–0.78]) but a 27% reduction in risk of death in the TPS < 1% group (median overall survival: 6.5 [95% CI 4.4–11.7] months vs. 5.5 [95% CI 3.7–8.5] months, HR 0.73 [95% CI 0.49–1.09]) [25]. Moreover, post hoc analyses in the CheckMate 141 revealed that ORR increased according to the intensity of TPS (17.0% in the TPS ≥ 1% group; 22.2% in the TPS ≥ 5% group; and 27.9% in the TPS ≥ 10% group) [4]. In our study, median PFS was similar in the CPS ≥ 1 and TPS ≥ 1% groups but was shorter in the TPS < 1% group than in the CPS < 1 group and significantly worse in the TPS ≥ 1% group. The CPS ≥ 1 group theoretically included all responders with TPS ≥ 1% but may have included more non‐responders because CPS quantifies both tumor cells and immune cells that express PD‐L1. We hypothesize that TPS might have potential to extract the non‐responders more accurately than CPS. Although there may have been some discrepancies in diagnosis according to the experience of the pathologists, incorrect training in calculation of CPS, background noise, biological heterogeneity, and other factors, the observer reproducibility for CPS is generally reasonable [26, 27, 28, 29, 30, 31]. However, interobserver reliability has been reported to be lower for intermediate groups and cases with values close to the cut‐off than for negative and strongly positive groups [31]. In this study, patients with positive CPS and negative TPS were mostly CPS 1 or 5 and had poor tumor responses, suggesting that TPS might be helpful as a negative indicator from the viewpoint of interobserver reliability (Table 4).

**TABLE 4:** Tumor response.

	All subjects, *N* = 53	Pembrolizumab, *n* = 26	Nivolumab, *n* = 27
Objective response rate, *n* (95% CI)			
Overall population	30.2 (19.5–43.5)	23.1 (11.0–42.1)	37.0 (21.5–55.8)
TPS < 1%	12.5 (2.2–47.1)	0.0	14.3 (2.6–51.3)
TPS ≥ 1%	33.3 (21.4–47.9)	24.0 (11.5–43.4)	45.0 (25.8–65.8)
CPS < 1	16.7 (3.0–56.4)	0.0	25.0 (4.6–70.0)
CPS ≥ 1	31.9 (20.4–46.2)	25.0 (12.0–44.9)	39.1 (22.2–59.2)
CPS ≥ 20	40.7 (24.5–59.3)	33.3 (15.2–58.3)	50.0 (25.4–74.6)
CPS ≥ 50	46.7 (24.8–69.9)	44.4 (18.9–73.3)	50.0 (18.8–81.2)
Clinical benefit rate, *n* (95% CI)			
Overall population	43.4 (31.9–56.7)	46.2 (28.8–64.5)	40.7 (24.5–59.3)
TPS < 1%	12.5 (2.2–47.1)	0.0	14.2 (2.6–51.3)
TPS ≥ 1%	48.9 (40.0–63.0)	48.0 (30.0–66.5)	50.0 (30.0–70.1)
CPS < 1	33.3 (9.7–70.0)	50.0 (25.6–63.2)	25.0 (4.6–70.0)
CPS ≥ 1	44.7 (31.4–58.8)	45.8 (27.9–64.9)	43.5 (25.6–63.3)
CPS ≥ 20	59.3 (40.7–75.5)	60.0 (35.7–80.2)	58.3 (32.0–80.7)
CPS ≥ 50	73.3 (48.0–89.1)	77.8 (45.3–93.7)	66.7 (30.0–90.3)
Best overall response (%)			
CR	3 (5.7)	1 (3.8)	2 (7.4)
PR	13 (24.5)	5 (19.2)	8 (29.6)
SD	11 (20.8)	9 (34.6)	2 (7.4)
Non‐CR/non‐PD	1 (1.9)	1 (3.8)	0
PD	23 (43.4)	9 (34.6)	14 (51.9)
NE	2 (3.8)	1 (3.8)	1 (3.7)

Abbreviations: CI, confidence interval; CPS, combined positive score; CR, complete response; NE, not evaluable; PD, progressive disease; PR, partial response; SD, stable disease; TPS, tumor proportion score.

This study faced several limitations. Firstly, it adopted a retrospective design at a single center with a restricted patient cohort. Additional studies in larger samples may lead to more suitable cut‐off values. Second, patients who received APA monotherapy tended to have less aggressive tumors and a relatively low tumor burden, suggesting selection bias. Therefore, the decision to use APA monotherapy should consider not only PD‐L1 expression but also tumor aggressiveness and the patient's overall health status. Third, regarding the tissue collection method, the surgical and biopsy samples were mixed, and the timing of collection was not consistent. PD‐L1 expression has been suggested to be heterogeneous, to differ by organ, and to change over time owing to the effects of radiotherapy and chemotherapy [32, 33]. Therefore, prediction of clinical outcomes may have been more accurate had more appropriate specimens for evaluation of PD‐L1 expression been available. Finally, this study assessed only the relationship between CPS and short‐term outcomes (i.e., CBR and PFS), and there is a need for further studies to identify an appropriate CPS cut‐off value for better prediction of long‐term outcomes.

In conclusion, our study indicated that CPS positivity varies according to tumor histology, and the CPS cut‐off value that predicts better clinical outcomes might be higher than in previous reports on R/M‐HNSCC patients treated with APA monotherapy. Based on the larger sample sizes, further investigations evaluating the utility of TPS to detect candidates for APA monotherapy are needed.

## Author Contributions

Yoshitaka Honma and Taisuke Mori were responsible for the study's conceptualization and methodology. Supervision was carried out by Yoshitaka Honma, Taisuke Mori, and Ken Kato. Project administration was handled by Yoshitaka Honma. All authors contributed to data curation, as well as to the review and editing of the manuscript. The final version of the manuscript was approved by all authors for submission.

## Ethics Statement

The study adheres to all applicable domestic regulations, institutional guidelines, and the ethical standards outlined in the Helsinki Declaration for research involving human subjects. In accordance with these principles, approval was obtained from the institutional review board or an equivalent ethics committee, and written informed consent was provided by all participants.

## Conflicts of Interest

The authors declare no conflicts of interest.

## Data Availability

The data supporting the findings of this study are available from the National Cancer Center Hospital, but these data are not publicly available. However, anonymized data can be obtained from the authors if the purpose of the data use is identified and specific requests and obtained permission from the National Cancer Center Hospital.
